# Supplementary figures and images for: Genome-Wide Association Studies for Flesh Color and Intramuscular Fat in (Duroc × Landrace × Large White) Crossbred Commercial Pigs
Source: Genes (Basel). 2022 Nov 16;13(11):2131. doi: 10.3390/genes13112131 (PMC9690869; doi:10.3390/genes13112131)

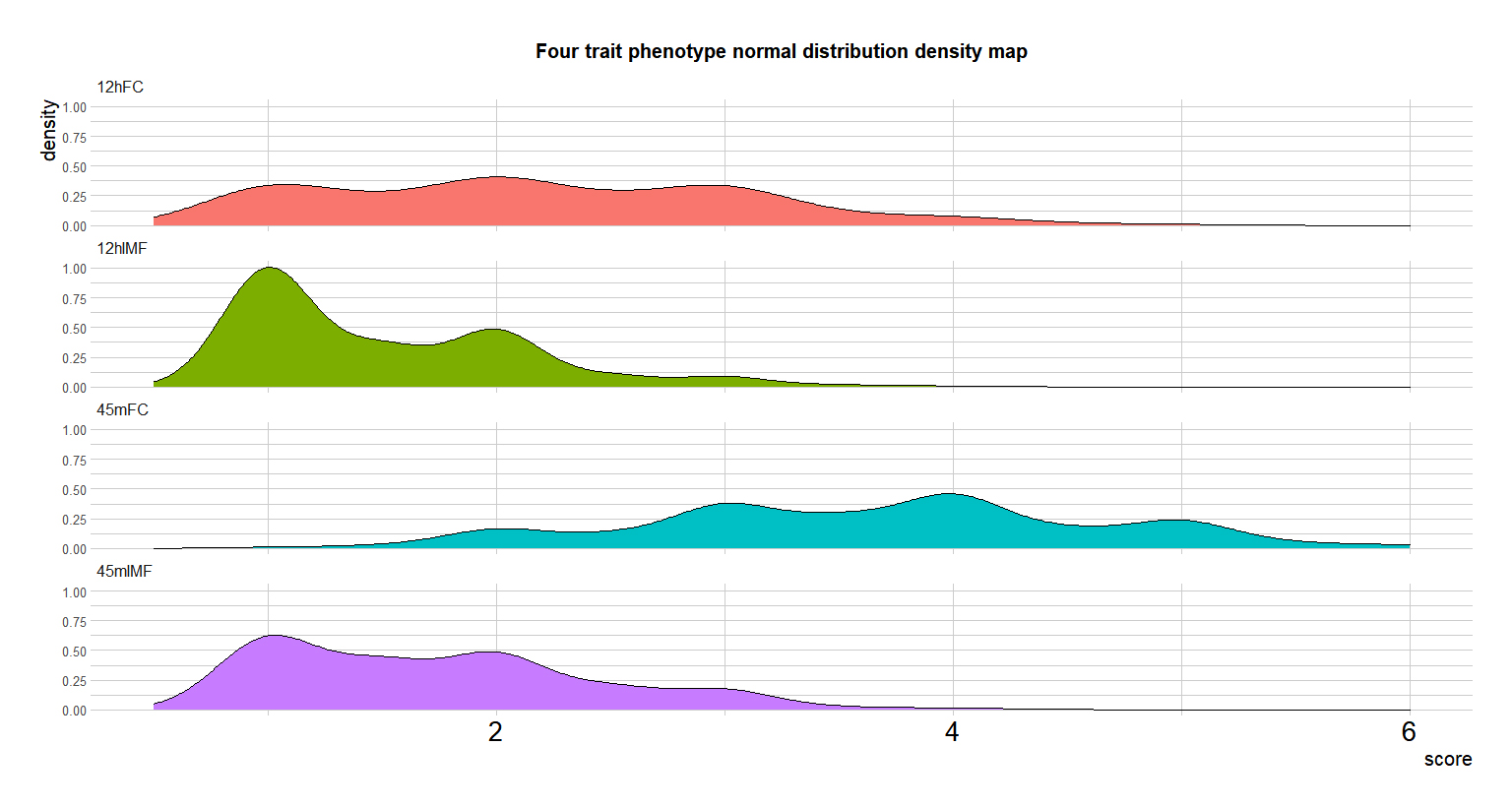

Supplement: Supplementary file 1 [file genes-13-02131-s001.zip › Figure S1.jpg]

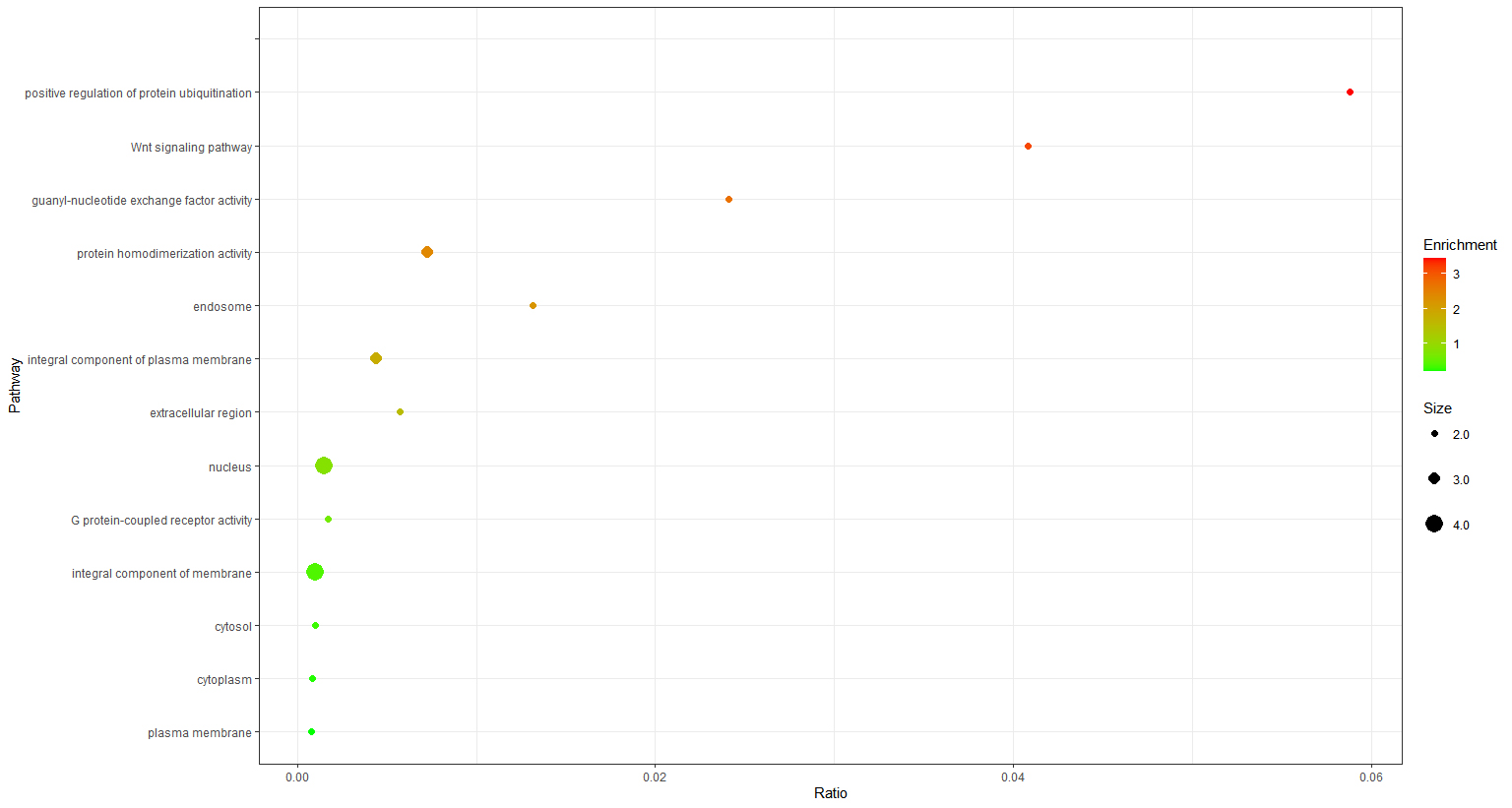

Supplement: Supplementary file 1 [file genes-13-02131-s001.zip › Figure S2.jpg]
